# An Inner City Emergency Medicine Rotation Does Not Improve Attitudes toward the Homeless among Junior Medical Learners

**DOI:** 10.7759/cureus.1748

**Published:** 2017-10-05

**Authors:** Aaron Sibley, Kathryn A Dong, Brian H Rowe

**Affiliations:** 1 Emergency Medicine, Dalhouse University, Halifax, NS.; 2 Department of Emergency Medicine, School of Community Based Medicine, University of Alberta

**Keywords:** emergency medicine, education, homeless

## Abstract

Introduction

Learners in the emergency department (ED) frequently encounter individuals who are homeless. We sought to evaluate the effect of an inner city emergency medicine rotation at the Royal Alexandra Hospital (RAH) on the attitudes of medical students and residents towards this population.

Methods

Data were collected both pre- and post-rotation using an electronic survey. Data collected included demographic information and as well as scores on the Health Professionals’ Attitudes Towards the Homeless Inventory (HPATHI). Pre- and post-survey results were compared using Wilcoxon tests.

Results

Ninety-eight students completed the rotation. A total of 40 (41%) students completed both pre- and post-surveys. Demographic information was available for 66 respondents. Most participants were male (42 {64%}), single (47 {71%}), and 30 years of age or younger (59 {89%}). Most participants were of a Caucasian or East/South Asian background (61 {92%}) and grew up in an urban setting (51 {77%}). Overall, 43 (90%) participants saw at least one person who was homeless during their rotation. There was no significant difference between pre- and post-aggregate scores (z = -0.78, p = 0.44), or any of its three subscales (Personal Advocacy, Social Advocacy, and Cynicism).

Conclusion

First year residents and medical students are frequently exposed to patients who are homeless during an inner city ED rotation. Attitudes towards these patients did not significantly change following the rotation. Educational objectives should be set to improve attitudes of learners towards those with unstable housing throughout the ED rotation.

## Introduction

Homelessness is endemic in urban North America. Obtaining an accurate estimate of the total number of homeless individuals is difficult; however, estimates suggest that approximately 35,000 Canadians and almost 550,000 individuals in the United States (US) are homeless on any given night [1-2]. Studies have shown that the homeless are frequent users of healthcare [3-5] and, in particular, the emergency department (ED) [4-12]. Use of EDs has been reported to be up to three times greater than the general public and up to 23% of the homeless report that the ED is their primary source of healthcare [3-4].

Despite the frequency of presentation, ED care of patients who are homeless can be fragmented and of poor quality [9,11]. One study has suggested that attitudes toward the homeless may be more negative among ED faculty than medical students [13]. Given the importance of presentations by patients who are homeless in the ED, we sought to examine the effect of an inner city emergency medicine rotation on the attitudes of medical students and first year residents toward the homeless population.

## Materials and methods

Setting

Senior medical students and first year medical residents completing clinical rotations at the Royal Alexandra Hospital (RAH) Emergency Department in Edmonton, Alberta were eligible to participate in the survey. The RAH is a large inner city teaching hospital and a Level two Trauma Center with 66,442 emergency visits between 2010-2011 and approximately 620 inpatient beds. The RAH serves many individuals with mental health diagnoses and substance use disorders who also face multiple social inequities such as a lack of a stable income and housing. Edmonton has a large urban indigenous population that is overrepresented in the ED population compared to other groups. Surveillance data indicates that 1.2% of ED patients at this site are infected with the human immunodeficiency virus (HIV) and 12% have hepatitis C virus (HCV) infections [14]. While the exact number of homeless patients who present to this ED is not systematically tracked, it has been estimated to be approximately 10% (K Dong 2011, personal communication).

Rotation

The clinical rotations at the RAH ED consisted of approximately three to four eight-hour shifts per week, over a two to four week period. Students and residents were paired with one physician per shift. Generally speaking, students and first year residents would independently see stable patients and then discuss investigation(s) and management plans with the staff physician. Aside from what was provided on an individual basis, there was no formal teaching about how to care for patients who are homeless in the ED.

Design

This study was a prospective cohort study with pre- and post-rotation electronic questionnaire completion. The study period was from July 1, 2008 to June 30, 2009.

Survey methods

Learners were asked to complete an electronic survey prior to and immediately following their inner city emergency medicine rotation. Surveys were distributed to the learners via email three weeks prior to the first day of their rotation with reminder notifications at one week and three days prior to the start of their rotation. Learners were asked to complete the same questionnaire again following the last shift of their rotation. The post-rotation survey was emailed to the learners three days after the rotation ended with reminder notifications at one and three weeks following the rotation. The survey took approximately 10 minutes to complete. Learners were made aware that participation in the study was voluntary and that proceeding to complete the survey implied consent. Learners were made aware that at anytime they could withdraw without penalty or adverse effects on their rotation evaluation.

Research tools

An electronic survey was created to collect information in four areas. First, demographic information was obtained. This included: age, sex, ethnic background, marital status, previous experience in an inner city emergency rotation, and province of upbringing. Second, learners were asked a series of eight 'yes/no' questions regarding the type of patients seen during their rotation. The next and largest part of the survey included the 19-item Health Professionals’ Attitudes Towards the Homeless Inventory (HPATHI) [15]. Representative items from the HPATHI are contained in Appendix 1.

The HPATHI was created for use by healthcare providers, to measure their attitudes toward the homeless. The design and validation of the HPATHI is described elsewhere [15]. The tool has demonstrated good internal consistency with a Cronbach’s alpha of 0.88 and a test-retest reliability of 0.69. A factor analysis revealed three potential subscales. These subscales represent: personal advocacy, social advocacy, and cynicism. Concurrent validity was examined by comparison of the respondent’s answers on a similar, previously validated tool, the Attitudes Towards Homelessness Inventory (ATHI) [16]. Pearson’s correlation coefficient between the HPATHI and the ATHI was 0.68 [15]. Finally, learners were given an opportunity to give feedback on their experience and suggest possible areas for improvement of the rotation.

Analysis

Data were analyzed using SPSS-PC statistical software (SPSS, IBM Corp., Armonk, NY). Continuous variables are reported as means with either standard deviations (SD) or percentage {%}, where appropriate. Overall scores on the HPATHI were non-normally distributed; a Wilcoxon test was conducted to evaluate whether there was a significant change in the students' attitudes towards the homeless pre- and post rotation. A multiple regression analysis was used to test if demographic variables significantly predicted participants’ HPATHI scores.

Ethics

This study protocol was approved by the Health Research Ethics Board of the University of Alberta.

## Results

Response

Of the 98 students who completed the rotation, 66 learners (medical students: 47 {71%}; residents: 19 {29%}) completed pre-rotation (n = 18), post-rotation (n = 8) or both (n = 40) questionnaires (response rate 67%). The mean time from completion of the pre- to the post-rotation questionnaires was 37.9 days (SD 10.3 days). Demographic information was available for 66 respondents (Table 1). Most participants were male (42 {64%}), single (47 {71%}), and 30 years of age or less (59 {89%}). Most participants were of Caucasian or East/South Asian background (61 {92%}) and grew up in an urban setting (51 {77%}). Three (4.5%) had completed a previous inner city hospital rotation.

**Table 1:** Description of learners participating in an inner city emergency medicine rotation.* *66 of 98 potential participants provided demographic information; SD = standard deviation.

	N (%) or Mean (±SD)
Level of Training:	
Medical Student	47 (71%)
First Year Resident	19 (29%)
Age (years):	26.8 (± 4.6)
Sex:	
Male	42 (64%)
Female	24 (36%)
Marital Status:	
Married/Common Law	19 (29%)
Single	47 (71.2%)
Ethnicity:	
White/Commonwealth/European	32 (49%)
East Asian (Chinese/Japanese/Korean)	16 (24%)
South Asian (Sri Lankan/East Indian)	13 (20%)
Other	5 (7%)
Upbringing:	
Urban	51 (77%)
Rural	15 (23%)

Contact with target groups

Of the participants who completed the post-rotation questionnaire, 43 (90%) had cared for a patient who was homeless, 46 (96%) had cared for a patient who was intoxicated, and 46 (96%) had cared for a patient who did not have a family physician. Overall, all learners encountered at least one patient from these groups.

Attitudes

The mean HPATHI score for pre-rotation was 70.45 and post-rotation was 70.96. A Wilcoxon test was conducted to evaluate whether there was a significant change in students' attitudes toward the homeless. The results (z = -0.78, p = 0.44) indicate that there was not a significant change in attitudes during the rotation. Further analysis of the three subscales did not show any significant change between pre- and post-rotation scores (Table 2). Multiple regression analysis was used to test if demographic variables (e.g., sex, marital status, level of training, and age) significantly predicted participants’ HPATHI scores. None of the variables (sex [p = 0.17]; marital status [p = 0.93], level of training [p = 0.79]; age [0.17]) were significant, and a robust model did not emerge (F4, 43 = 0.969, p = n.s.), R2 = .08.

**Table 2:** Scores on the Health Care Providers Attitudes Towards the Homeless Inventory (HPATHI) before and after an emergency department rotation ^1^ p-values calculated using the Wilcoxon Signed Ranks Test; SD = standard deviation.

Scale	Pre-Rotation Score (Mean ± SD)	Post-Rotation Score (Mean ± SD)	p-value^1^
Personal Advocacy	38.0 (± 5.0)	38.4 (± 5.5)	0.41
Social Advocacy	22.9 (± 1.7)	23.1 (± 2.0)	0.47
Cynicism	7.65 (± 1.1)	7.45 (± 1.2)	0.25
Total Score	70.45 (± 6.5)	70.96 (± 7.1)	0.44

Qualitative data

Although feedback was sought from learners following the elective about their experience, overall, there were few comments. Those who responded, however, commented on how they appreciated the varied patient population and patient presentations, in particular the exposure they received to vulnerable and marginalized patients. When asked about ways to improve the rotation, one student commented that a short orientation package of social services and community resources would have been helpful (Table 3).

**Table 3:** Selected comments from anonymous medical student and resident learners in an inner city rotation. ED = emergency department; GP = general practitioner.

What did you like about your emergency medicine rotation at the Royal Alexandra Hospital?	Is there anything you would add or change regarding your rotation at the Royal Alexandra Hospital?
Nice mix of patients. Exposure to marginalized and underserved populations.	Saw a lot of chronic patients that did not have a GP
I liked the wide range in the patient population, and the challenges of their complex medical problems.	An orientation package on social services, community kitchens or other support services for different cultural groups and for homeless individuals
Physician attitudes towards marginalized populations is exceptional compared to other EDs that I have seen.	
Respect shown to those in need despite level of intoxication or even when belligerent.	

## Discussion

Patients who are homeless frequently present to the ED in this Canadian center and they often identify the ED as the primary source of health care. The lack of health care continuity, a chaotic lifestyle, environmental exposures, and food insecurity leave patients who are homeless vulnerable to a variety of medical complications, many of which are best suited for management in an ED setting.

Caring for a patient who is homeless (or marginally housed) can be one of the most challenging situations faced in a busy ED. Unfortunately, patients who are homeless may be seen as “difficult” or “problem” patients who abuse emergency medical services transport systems and the ED. More than half of the respondents on our pre-survey felt overwhelmed by the complexity of the problems experienced by patients without stable housing. In this regard, it is not surprising that patients who are homeless may feel unwelcome or discriminated against when accessing healthcare [17]. Up to 40% of homeless people report being judged unfairly, or treated with disrespect by a doctor or medical staff at least once in the last year [18]. Many people who are homeless feel healthcare workers lack compassion [19].

Our study demonstrates that attitudes toward homeless patients did not change following a rotation in a busy inner city ED. The program was limited to a two to four week rotation without any structured lectures or community homelessness interactions. These results appear to be similar to some other research efforts. For example, a 2009 study did not show a significant change in attitudes following a family medicine rotation in a Homeless Care Clinic in West Virginia [20]. Unfortunately, one paper examining the attitudes of British medical students toward the homeless reported that attitudes deteriorated (e.g., became significantly more negative) following their clinical years [21].

Conversely, some interventions have resulted in positive changes. Two studies evaluating the effect of a two-week rotation in homeless health for family medicine residents in Chicago demonstrated improvements in attitudes toward patients who are homeless [22-23]. So too did a homeless outreach clinic experience for family nurse practitioner students [24]. These studies were unique in that students participated in a formal curriculum on the homeless including theory, clinical experiences, and role modeling. Finally, dental students rotating through a dental clinic for the homeless also showed an improvement in attitudes towards the homeless post rotation [25]. The dental students worked closely with an assistant who often knew the patient and was confident in managing both behavior issues and the patient’s specific oral health problem.

There are likely many reasons for these mixed results including the target educational groups (e.g., medical student, interns, residents), the generally privileged background of most medical students [26], the clinical site (e.g., clinic vs. ED), the educational curriculum (e.g., video, didactic, community involvement, etc), exposure to non-medical doctor (MD) staff (e.g., interpreters, social workers, case managers, addiction experts, etc) and the tool(s) used to evaluate the learners. In our study, we targeted learners early in their training, at one high-volume inner city ED, and did not provide any structured training about caring for patients who are homeless. Their training was exclusively ED MD focused, and no other health care professionals were involved. Of note, while scores in our study on both the personal advocacy and social advocacy subscales improved slightly after the rotation, scores on the cynicism scale worsened slightly suggesting that learners may have become more cynical towards this population.

Preparing medical learners to meet the needs of this vulnerable group is critical if the health system wishes to respond effectively to their needs. Training non-medical volunteers to provide “compassionate care” including attentive listening and food in the ED has shown promise in decreasing the number of visits [27]. Given the high intelligence and motivation shared by most medical students and residents, it is reasonable to suggest that they also could be trained to provide more “compassionate care” to patients who are homeless.

Study limitations

This study has several important limitations: it is limited by a relatively small sample size and incomplete rate of participant response. The date of these rotations was between 2008 and 2009. It is possible that the attitudes of current learners have changed since this time; however, at this institution there is still no structured teaching or intervention regarding care for individuals who are homeless and the patient demographics have not changed. Interestingly, 10% of learners stated they did not care for a homeless person on their rotation. Given the changing diversity of the homeless population, it is possible that some learners did not recognize a patient was in fact homeless [1]. Learner rotations were of two to four weeks duration, which may not have been a sufficient exposure to this population to result in an attitudinal change. Also, a clinically important difference in scores or a cut-off score indicating an “acceptable” attitude level for health care professionals has not been reported for the HPATHI. Finally, despite assurance of anonymity, responders may have been unduly influenced to select the more socially acceptable responses to questions rather than their actual beliefs.

## Conclusions

In conclusion, while learners were frequently exposed to homeless individuals and/or intoxicated patients during their rotation, their attitudes toward this group did not change significantly during an MD-based inner city ED rotation. Exposure to homeless patients alone may not be sufficient to change attitudes and further educational interventions during ED rotations may be warranted. Future work should evaluate more formal curricula tailored to an ED setting.

## Appendices

Appendix 1: Sample items from each subscale of the HPATHI

Personal Advocacy

Doctors should address the physical and social problems of the homeless.

I entered medicine to help those in need.

Social Advocacy

Homeless people are victims of circumstance.

Homeless people have the right to basic healthcare.

Cynicism

Homeless people are lazy.

Healthcare dollars should be directed toward serving the poor and homeless.
